# Bactericidal and biofilm eradication efficacy of a fluorinated benzimidazole derivative, TFBZ, against methicillin-resistant *Staphylococcus aureus*

**DOI:** 10.3389/fphar.2024.1342821

**Published:** 2024-04-10

**Authors:** Qian Chen, Zhihui Dong, Xuedi Yao, Huan Sun, Xin Pan, Jikai Liu, Rong Huang

**Affiliations:** ^1^ The Modernization Engineering Technology Research Center of Ethnic Minority Medicine of Hubei Province, School of Pharmaceutical Sciences, South-Central Minzu University, Wuhan, China; ^2^ International Cooperation Base for Active Substances in Traditional Chinese Medicine in Hubei Province, School of Pharmaceutical Sciences, South-Central Minzu University, Wuhan, China

**Keywords:** methicillin-resistant *Staphylococcus aureus*, biofilms, benzimidazole derivative, antibacterial, biofilm eradication

## Abstract

Methicillin-resistant *Staphylococcus aureus* (MRSA) is a major inducement of nosocomial infections and its biofilm formation render the high tolerance to conventional antibiotics, which highlights the requirement to develop new antimicrobial agents urgently. In this study, we identified a fluorinated benzimidazole derivative, TFBZ, with potent antibacterial efficacy toward planktonic MRSA (MIC = 4 μg/mL, MBC = 8 μg/mL) and its persistent biofilms (≥99%, MBEC = 8 μg/mL). TFBZ manifested significant irreversible time-dependent killing against MRSA as characterized by diminished cell viability, bacterial morphological change and protein leakage. Furthermore, the results from CBD devices, crystal violet assay in conjunction with live/dead staining and scanning electron microscopy confirmed that TFBZ was capable of eradicating preformed MRSA biofilms with high efficiency. Simultaneously, TFBZ reduced the bacterial invasiveness and exerted negligible hemolysis and cytotoxicity toward mammalian cells, which ensuring the robust therapeutic effect on mouse skin abscess model. The transcriptome profiling and quantitative RT-PCR revealed that a set of encoding genes associated with cell adhesion, biofilm formation, translation process, cell wall biosynthesis was consistently downregulated in MRSA biofilms upon exposure to TFBZ. In conclusion, TFBZ holds promise as a valuable candidate for therapeutic applications against MRSA chronic infections.

## Graphical Abstract

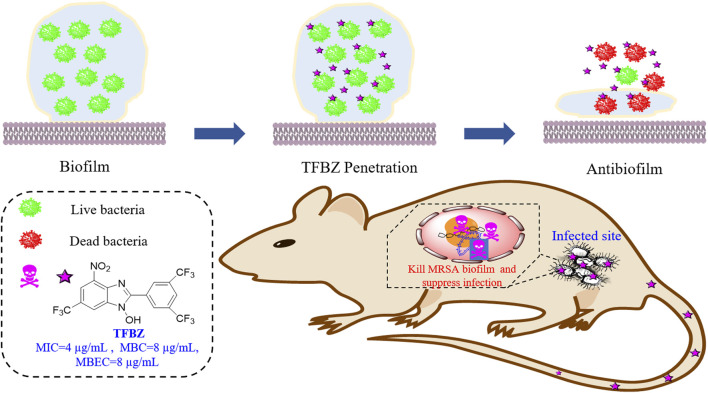

## 1 Introduction

The widespread abuse of antibiotics in medicine, agriculture, and veterinary practices have led to their accumulation in freshwater sources and wastewater, becoming a notable contaminant. Millions of tons of these emerging pollutants are discharged into aquatic environments annually, fostering the emergence of drug-resistant genes and bacteria (Kaur Sodhi and Singh, 2022; Sodhi et al., 2023). Concurrently, the COVID-19 pandemic has potentially diminished the efficacy of certain medications against specific pathogens (Singh and Sodhi, 2023). Consequently, there is heightened concern about multidrug-resistant (MDR) bacteria, especially in bacterial communities frequently exposed to antibiotics (Xu et al., 2021). In recent decades, MDR bacteria such as methicillin-resistant *Staphylococcus aureus* (MRSA) and methicillin-resistant *Staphylococcus epidermidis* (MRSE) have become predominant in causing persistent infections across hospitals, communities, and livestock, leading to severe health issues like sepsis, pneumonia, and endocarditis with considerable morbidity and mortality (Hu et al., 2017; Qin et al., 2023).

Bacterial multidrug resistance emerges not only from the structural transformation or genetic mutation of planktonic bacteria but also from the biofilms formed by bacterial persister cells. Biofilms are three-dimensional communities of aggregated bacterial cells enveloped in self-produced extracellular polymeric substances (EPS), adhering irreversibly to both biotic and abiotic surfaces, thus facilitating evasion from the host immune response and enhancing tolerance to antimicrobial treatments (Cui et al., 2020; Chen et al., 2023). Remarkably, bacteria within biofilm exhibit antibiotic resistance that is 10 to 1,000 times greater than their planktonic counterparts (Shree et al., 2023).

Investigations into MRSA biofilm production have uncovered a complex signaling network regulating the upregulation and downregulation of adhesion genes involved in biofilm assembly and virulence factor synthesis throughout infection stages. This regulatory mechanism acts as a genetic seesaw to express the pivotal components for biofilm stability and MRSA pathogenesis including intercellular adhesin, extracellular DNA, wall teichoic acids, and so on (Patel and Rawat, 2023). The secretion of diverse virulence factors, like hemolysins, leukotoxins, enterotoxins, and Protein A, is regulated by agr and sae two-component systems, playing a crucial role in combating the host immune response. Regulatory factors such as the Agr regulatory system, the SarA protein family, the transcription factor σB, and the signal transduction system LuxS/AI-2 are involved in the quorum sensing system, the secretion of virulence factors, and biofilm dispersal and dissociation (Balamurugan et al., 2017). Moreover, genes like *atlE*, *bap*, *sasG*, and *ica* regulate the surface adhesion and cell accumulation (Yang et al., 2016).

At present, numerous approaches have been put forward to combat drug-resistant bacteria biofilms, involving antimicrobial peptides (AMPs), naturally derived compounds, and growth-neutral dispersants (Singh et al., 2023). Research into agents capable of eradicating biofilms is accelerating, with a focus on those that enhance existing therapeutic methods (Garrison et al., 2016; Deusenbery et al., 2023). Innovative non-chemotherapeutic approaches like photodynamic and photothermal therapy are also under exploration for their biofilm-disrupting potential. AMPs are particularly noted for their ability to disrupt bacterial cell membranes, thereby impeding biofilm formation. RN3, a natural AMP, is distinguished for its ability to permeabilize biofilm cells, highlighting the broader utility of AMPs in targeting microbial biofilms (Srinivasan et al., 2021). Compounds from amphibian skin secretions, akin to AMPs, have shown efficacy against various biofilm-forming pathogens. Japonicin-2LF from the skin secretion of the Fujian large-headed frog (*Limnonectes fujianensis*) not only disrupts MRSA biofilms by targeting the membrane but also effectively clears both planktonic and sessile bacterial populations, representing a promising approach for treating MRSA in cystic fibrosis patients (Yuan et al., 2019). However, the clinical application of AMPs for biofilm inhibition faces challenges, primarily due to their degradation by bacterial proteases, which affects their stability and efficacy *in vivo*. This issue underscores the necessity for more potent anti-biofilm agents and has spurred interest in bioactive compounds from alternative sources, particularly medicinal plants. Extracts from Indian medicinal plants like *Cinnamomum glaucescens* and *Syzygium praecox* are being explored for their anti-biofilm activities against *S. aureus* (Panda et al., 2020). Phytochemicals such as 12-Methoxy-trans-carnosic acid and carnosol, found in *Salvia officinalis*, have demonstrated potent *in vitro* anti-biofilm effects against *Candida* species (Kerkoub et al., 2018). Moreover, myrtenol, a monoterpenoid from certain plants, has shown efficacy in targeting the *sarA* gene, thereby inhibiting MRSA biofilm formation and highlighting the potential of plant-derived compounds as alternative therapeutic agents against biofilm-associated infections (Selvaraj et al., 2019). Despite these advancements, the journey towards FDA approval for these compounds remains challenging, with many promising candidates faltering in clinical trials due to non-selectivity, cytotoxicity, or hemolysis risks (Lu et al., 2019). Additionally, potential adverse effects such as inflammatory responses and oxidative DNA damage pose significant obstacles to their clinical utility (Yu et al., 2022).

**FIGURE 1:**
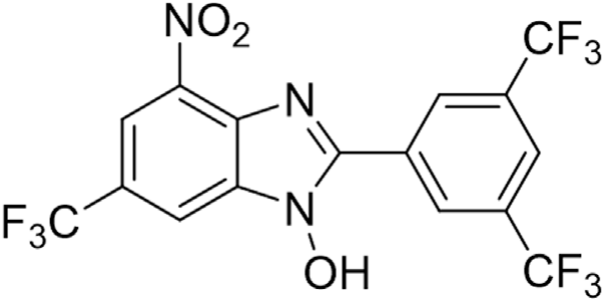
The chemical structure of TFBZ.

Considering the abundant secondary metabolites of fungi are important resources for mining natural antimicrobial agents, it stands to reason that such sources and derivates are fertile grounds for the discovery of biofilm-eradicating agents. Here in this work, we screened from the long-accumulated library of diverse fungal natural products and pharmacophore-like compounds in our group, fortunately identified a fluorinated benzimidazole analogue TFBZ (2-(3,5-Bis(trifluoromethyl)phenyl)-4-nitro-6-(trifluoromethyl)-1H-benzo [d]imidazol-1-ol, the structure is illustrated in Figure 1), which displays outstanding antimicrobial activity and potent biofilm eradication property against MRSA. This compound was synthesized according to a catalytic trifluoromethoxylation method reported by Zheng et al., to finally get a quantity of 531 mg included in our library (Zheng et al., 2018). Benzimidazole derivatives, similar in structure to natural nucleosides, act as key intermediates in the synthesis of new chemical entities with biological interest. Previous literatures reported that benzimidazole derivatives possess extensive pharmacological activities as DNA binding agents, enzyme inhibitors (Zha et al., 2021), anti-malarial agents and biofilm-eradicating agents (Kong et al., 2018). Our comprehensive research aims to thoroughly investigate the *in vitro* and *in vivo* efficacy of TFBZ against both planktonic and persistent MRSA, shedding light on its potential mechanism of action and paving the way for novel therapeutic approaches against antibiotic-resistant bacterial infections.

## 2 Materials and methods

### 2.1 Synthesis of 2-(3,5-Bis (trifluoromethyl)phenyl)-4-nitro-6-(trifluoromethyl)-1H-benzo [d]imidazol-1-ol (TFBZ)

2-(3,5-Bis (trifluoromethyl)phenyl)-4-nitro-6-(trifluoromethyl)-1H-benzo [d]imidazol-1-ol (TFBZ) was synthesized following an existing procedure (Zheng et al., 2018). Yellow solid, 531 mg, 74% yield. ^1^H-NMR (500 MHz, DMSO-*d*_6_) *δ* 8.70 (s, 2H), 8.24 (d, *J* = 15.9 Hz, 2H), 8.12 (s, 1H); ^13^C-NMR (125 MHz, DMSO-*d*_6_) *δ* 149.4, 138.4, 136.5, 133.1, 131.2 (q, *J* = 33.4 Hz), 129.8, 128.9, 124.9, 123.9 (q, *J* = 271.3 Hz), 123.3 (q, *J* = 271.3 Hz), 123.1 (q, *J* = 33.8 Hz), 116.4 (q, *J* = 2.5 Hz), 113.8(q, *J* = 2.5 Hz). ^19^F NMR (471 MHz, DMSO-*d*_6_) *δ* −59.63 (s, 3F), −61.67 (s, 6F). HRMS (ESI): Calcd for: C_16_H_7_F_9_N_3_O_3_^+^ ([M + H] ^+^) 460.0338, found: 460.0334.

### 2.2 Strains and cultural conditions

The bacterial strains used in the present study were *E. coli* (*E. coli,* ATCC 25922), *S. aureus* (*S. aureus*, ATCC 25923) and methicillin-resistant *S. aureus* (MRSA, ATCC 43300), *P. aeruginosa* (*P. aeruginosa*, ATCC27853) and *Salmonella enterica subsp. enterica* (*Salmonella*, ATCC14028). MRSA, *P. aeruginosa* and *Salmonella* was provided by Kunming Institute of Botany, Chinese Academy of Sciences (Kunming, China). *S. aureus* and *E. coli* were obtained from the microbial genetics’ laboratory, Wuhan University (Wuhan, China). Each bacterial strain was preserved in 30% glycerol solution (v/v) and frozen at −80°C. Before each experiment, the test strain was shake-cultured overnight at 37°C in medium (Luria–Bertani broth (LB) for *S. aureus* and *E. coli*, tryptic soy broth (TSB) for MRSA, Mueller-Hinton Broth (MHB) for *P. aeruginosa*, and *Salmonella*), and the bacterial suspensions in the mid-log phase (OD_600_ around 0.6–0.8) were harvested by centrifugation at 3,000 rpm for 10 min.

### 2.3 Minimum inhibitory concentration (MIC) and minimum bactericidal concentration (MBC) determination

Microtiter broth dilution testing was employed to detect the MIC following Clinical Laboratory Standards Institute (CLSI) M100-31st guidelines (Humphries et al., 2021). Briefly, 100 μL (10^4^ CFU/mL) bacterial suspensions and equal volume of 2-fold serial diluted TFBZ or vancomycin were added into sterile 96-well plates and incubated at 37°C for 18 h, and then the bacterial viability was determined by OD_600_ using a microplate spectrophotometer (Spark 10 M, Tecan, Switzerland). Subsequently, the pretreated bacterial suspension was diluted at 1:10,000 and spread on agar plate medium for another 24-h incubation, then the CFUs were photographed and quantitatively analyzed. The MIC was deemed as the lowest sample concentration completely inhibiting the growth of visible microorganisms in the wells, the MIC_50_ was supported by measuring the turbidity at OD_600_ and the MBC was determined by observing no visible colony formation on the agar plate (Zhang et al., 2020). The concentration range of TFBZ tested during this study was 1–8 μg/mL for Gram-positive *S. aureus* and MRSA, 1–64 μg/mL for Gram-negative *E. coli*, *P. aeruginosa*, and *Salmonella*. Untreated and vancomycin-treated bacterial suspension were regarded as negative and positive control, respectively.

### 2.4 Time-dependent killing assay

MRSA suspension (10^4^ CFU/mL) was co-cultured with varying final concentrations of TFBZ or vancomycin (0.5–8 μg/mL) in TSB broth at 37°C. At the designated time points of 0, 2, 4, 6, 12, 18, and 24 h, the cell growth was recorded at OD_600_ (Kang et al., 2020). After 24-h stationary culture, MTT was added in each well with the final concentration of 0.5 mg/mL, followed by 4-h incubation. Then, the supernatant was discarded and DMSO was added to dissolve the resultant crystal (Xu et al., 2023). The absorbance at 570 nm was measured with a microplate reader and MRSA viability was calculated as the following formula: MRSA viability (%) = [(OD_blank_–OD_sample_)/OD_blank_] × 100%.

### 2.5 Bacterial protein leakage determination

Typically, the bacterial suspensions were transferred to the sterile Eppendorf tubes which contained 2-fold serial dilutions of the test compounds (final concentrations: 0, 1, 2, and 4 μg/mL) and incubated at 37°C with 160 rpm shaking. Equal volume of bacteria mixture was taken out at different time intervals (0, 4, 8, and 12 h) and centrifuged for 10 min. The protein content was determined by the Enhanced BCA Protein Assay Kit (Beyotime, Shanghai, China) and corresponding protein concentration was extrapolated from the equation of best-fit linear regression line of the BCA standard curve (Liu et al., 2023).

### 2.6 Biofilm eradication assay

The minimum biofilms eradication concentrations (MBECs) of TFBZ and control compound vancomycin against MRSA were evaluated using the Calgary Biofilms Device (CBD) [Innovotech, product code: 19111)] as described previously (Garrison et al., 2016). In brief, MRSA biofilms were established on pegs that are submerged in inoculated mid log phase bacterial suspension overnight in 96-well plates. Subsequently, the CBD lid with forming biofilms was collected, washed twice by PBS, and transferred to a challenge plate which was contained serial dilutions of the test compounds and incubated for 24 h to dislodge biofilms. To determine MBEC values, CBD lid with remaining persisters was transferred to a recovery 96-well plate containing 200 μL nutrient TSB medium and incubated overnight at 37°C. The MBEC value is interpreted as the lowest concentration leading to the eradication of the biofilms (no turbidity after the final incubation period).

The biofilm eradication capability of TFBZ was further evaluated by crystal violet staining. Concisely, 200 μL bacterial suspension (10^5^ CFU/mL) was transferred to a sterile 96-well plate and incubated for 48 h statically for cell attachment to the surface. Subsequently, the forming biofilms were washed three times with PBS and cultivated with TFBZ or vancomycin for another 24 h at the concentration ranging from 1 to 8 μg/mL. After methanol fixation, 150 μL of 1% (v/v) crystal violet solution (Servicebio, Wuhan, Hubei, China) was added for 15min, then rinsed away redundant dye with sterile water and dried naturally. In addition, residual biofilms were further destained in 33% acetic acid for 20 min and quantified at 595 nm by using a microplate reader. The amount of biofilm was directly proportional to the OD value of the crystal violet solution in wells (Jiang et al., 2022). The clearance rate of biofilms was calculated as following formula: Biofilms clearance rate (%) = [1-(OD _Sample_/OD _PBS_)] × 100%

### 2.7 Scanning electron microscopy (SEM)

The bacterial suspension (10^8^ CFU/mL) was cultured in 6-well plates with coverslips (d = 1.0 cm) at 37°C for 24 h. The cover-glasses were then rinsed gently with sterile PBS and treated with 0, 4, and 8 μg/mL of TFBZ for 4 h and incubated without shaking at 37°C. Subsequently, samples were washed with PBS twice and submerged in a 2.5% glutaraldehyde solution for fixation overnight. After gradual dehydration with gradient ethanol (30%, 50%, 70%, 80%, 90%, and 100%) for 10 min each, the biofilms were freeze-dried and sputter-coated with gold (Huang et al., 2022). Finally, the morphology of specimens was imaged under a scanning electron microscope (Hitachi SU8010, Japan).

### 2.8 Live/dead staining

LIVE/DEAD BacLight bacterial viability kit (Bestbio, Shanghai, China)) was used to investigate the viability of the planktonic bacteria upon TFBZ treatment. In short, 1 mL of MRSA suspension (10^7^ CFU/mL) was seeded in confocal dishes and cultured with different final concentrations of TFBZ (0, 4, and 8 μg/mL) over 2 h at 37°C, respectively. After treatment, the bacterial suspensions were centrifuged at 3,000 rpm for 5 min, washed twice and re-suspended with saline, and then stained with SYTO9 (N01) (λ_ex_ = 500 nm, λ_em_ = 525 nm) and propidium iodide (PI) (λ_ex_ = 535 nm, λ_em_ = 615 nm) for 30 min at room temperature (Zhang et al., 2021). The fluorescence of each sample was observed by Confocal laser scanning microscopy (Carl Zeiss LSM 900, Germany)

To determine the viability of persistent biofilms, the mature biofilms were developed statically at 37°C for 24 h on glass coverslips set in six-well plates. After removing the unbound cells, the coverslips were treated with TSB alone or TFBZ (4 and 8 μg/mL) for another 4 h. Then the coverslips were gently rinsed with PBS, followed by staining with N01 and PI in darkness for 30 min, and imaged on Confocal in Z-axis scan mode (Zhang D.-Y. et al., 2023).

### 2.9 Adhesion and infection assays

The adhesion and infection experiments were performed as described by Emeri and co-authors (Emeri et al., 2019) with minor modifications. Breast cancer cells (MCF-7) obtained from China Center for Typical Culture Collection (CCTCC, Wuhan, China), were seeded in a 6-well plate and incubated under a humidified 5% CO_2_ to form monolayer cells. For the adhesion assay, aliquots of 1 mL of MRSA solution containing different concentration of TFBZ (ranging from 0 to 8 μg/mL) were transferred to each well and co-cultivated with MCF-7 cells at 37°C for 2 h. After repeated rinse with sterile PBS, MCF-7 cells were harvested and lysed with 0.1% Triton X-100. Finally, 100 µL of diluted lysis was coated onto the TSB agar plate, and incubated overnight to evaluate the number of forming colonies. For the invasion assay, after 2 h bacterial infection (same methods and conditions as above), MCF-7 cells were cultured with DMEM medium containing gentamycin for another hour (Tan et al., 2016). The remaining procedure was the same as that for the adhesion test.

### 2.10 Hemolysis and cytotoxicity assay

100 μL of Fresh rabbit red blood cells (rRBCs, 8%, v/v) was incubated with different concentrations of TFBZ (0.78–100 μg/mL) for 2 h at 37°C and centrifuged at 3,000 rpm for 5 min. After photographing, the absorption of the supernatant was measured by a microplate reader at 572 nm. PBS (0% hemolysis) and deionized water (DI, 100% hemolysis) were set as the negative and positive control, respectively (Kim et al., 2022). The hemolysis was calculated as following formula: Hemolysis (%) = (OD _Sample_−OD _pbs_)/(OD _DI_−OD _pbs_) ×100%

*In vitro* cytotoxicity of TFBZ was evaluated against African green monkey kidney fibroblast cells (COS 7, also obtained from CCTCC) using MTT method. COS 7 cells were planked in 96-well plated at a density of 1 × 10^4^ cells/well. Subsequently, COS 7 cells were cultivated by replaced medium containing different concentrations of TFBZ (0.78–100 μg/mL) for 24 h at 37°C. 20 μL of MTT (5 mg/mL in PBS) was pipetted in and further co-cultured with cells for 4 h. Finally, the supernatant was replaced by dimethyl sulfoxide to dissolve the purple formazan crystal and the cell viability was calculated on the basis of the absorbance at 568 nm (Li et al., 2020). The cell viability was calculated as following formula: Cell viability (%) = (OD _Sample_/OD _PBS_) ×100%

### 2.11 Skin abscess model

Animal experiments were approved by the Animal Ethics Committee of South-Minzu University for Nationalities (SYXK(Wuhan) 2016−0089, No. 2021-SCUEC-048) and performed in accordance with the Committee’s guidelines. Forty 5-week-old female BALB/c mice were purchased from Liaoning Changsheng Biotechnology Co., Ltd. (Certificate SCXK 2020−0001; Liaoning, China). Mice were pretreated with cyclophosphamide, at doses of 150 mg/kg for 96 h and then 100 mg/kg for 24 h, leading to the neutropenia and hypoimmunity. Subsequently, each mouse was subcutaneously injected with 100 μL of MRSA (10^8^ CFU/mL) into the right flank, initiating an infection that was allowed to develop for 36 h before therapeutic intervention. After 36 h of infection, mice were randomly divided into four groups (*n* = 8): (i) Model (treated with PBS vehicle), (ii) Vancomycin (10 mg/kg), (iii) low-dose (10 mg/kg TFBZ), and (iv) high-dose (20 mg/kg TFBZ). In addition, a normal control group was included, which received no treatment. Subsequently, the corresponding TFBZ doses were administered twice via the caudal vein. The mice were weighed and scored every 24 h, and then sacrificed after 9 days of therapy. The mice were weighed and clinically scored every 24 h according to the established standards in previous literature (Herrero et al., 2011). The infected subcutaneous tissues were excised and homogenized in normal saline. The samples were then serially diluted and cultured on LB agar for 24 h, followed by the CFUs. Furthermore, the main organs were collected and evaluated for *in vivo* toxicity using hematoxylin and eosin (H&E) staining.

### 2.12 RNA sequencing (RNA-seq) and reverse transcription-quantitative PCR (RT-qPCR) validation

MRSA ATCC 43300 biofilms were cultured as described in the previous section, resulting in the formation of two groups (*n* = 3): TFBZ-treated group with a concentration of 1 μg/mL (1/4 MIC value) and control group with the same volume of DMSO (vehicle). Biofilm cells were harvested 24 h post-treatment, and total RNA was extracted using a Tiangen Biotech kit (Beijing, China). Subsequently, RNA quantification was determined on Qubit^®^ 2.0 Fluorometer (Invitrogen, Grand Island, NY), and the quality control was performed using the Agilent 2,100 Bioanalyzer (Agilent Technologies, Inc.). The high-quality RNA was used for library preparation and Illumina sequencing, which was conducted at Berry Genomics Co., Ltd. (Beijing, China). Gene expression levels were quantified using fragments per kilobase of transcript per million mapped reads (FPKM) metrics, and differentially expressed genes (DEGs) was rigorously assessed utilizing HT-seq and edgeR algorithms. Statistical significance was determined by employing a false discovery rate (FDR) threshold of less than 0.05 and an absolute log2 fold change greater than 1. To elucidate the biological impact of TFBZ on MRSA, Kyoto Encyclopedia of Genes and Genomes (KEGG) functional enrichment analysis were employed to identify DEGs that were significantly enriched in metabolic pathways (Kanehisa and Goto, 2000; Kanehisa, 2019; Kanehisa et al., 2023).

Moreover, real-time PCR was employed to validate RNA-seq results by detecting select gene transcripts associated with the biofilm formation. Specifically, the analysis focused on genes including *sdrC*, *clfB*, *infC*, *dltB*, *groEL*, *murQ*, *moeA*, *rplN*, *icaD*, and *icaA*. The 16S rRNA was chosen as internal reference and the corresponding primer sequences were designed in Primer Premier v5.0 and synthesized by Tsingke Biotechnology Co., Ltd (Supplementary Table S3). Briefly, total RNA was isolated from MRSA biofilms treated and untreated with TFBZ, and then the complementary DNA (cDNA) was synthesized using the cDNA Synthesis Kit (Thermo Fisher, United States) according to the manufacturer’s guidelines. Subsequently, qPCR assays were conducted in triplicate on an ABI 7500 system (Thermo, United States), using SYBR Green Master Mix (Thermo Fisher, United States). The thermal cycling conditions were initiated with a 2-min denaturation at 95°C, followed by 40 cycles of 15-s denaturation at 95°C and a 30-s extension at 60°C, and a subsequent melting curve analysis was executed under specified conditions. The internal reference gene was 16s rRNA. Relative gene expression changes were calculated using the 2^^−∆∆CT^ method.

### 2.13 Statistical analysis

All data were expressed as the mean ± standard deviation (SD). Multiple comparisons were analyzed using one-way analysis of variance (ANOVA) followed by Tukey’s *post hoc* test, and *p*-value <0.05 was considered to be statistically significant.

## 3 Results and discussion

### 3.1 Antimicrobial activities of TFBZ against planktonic growth

**FIGURE 2:**
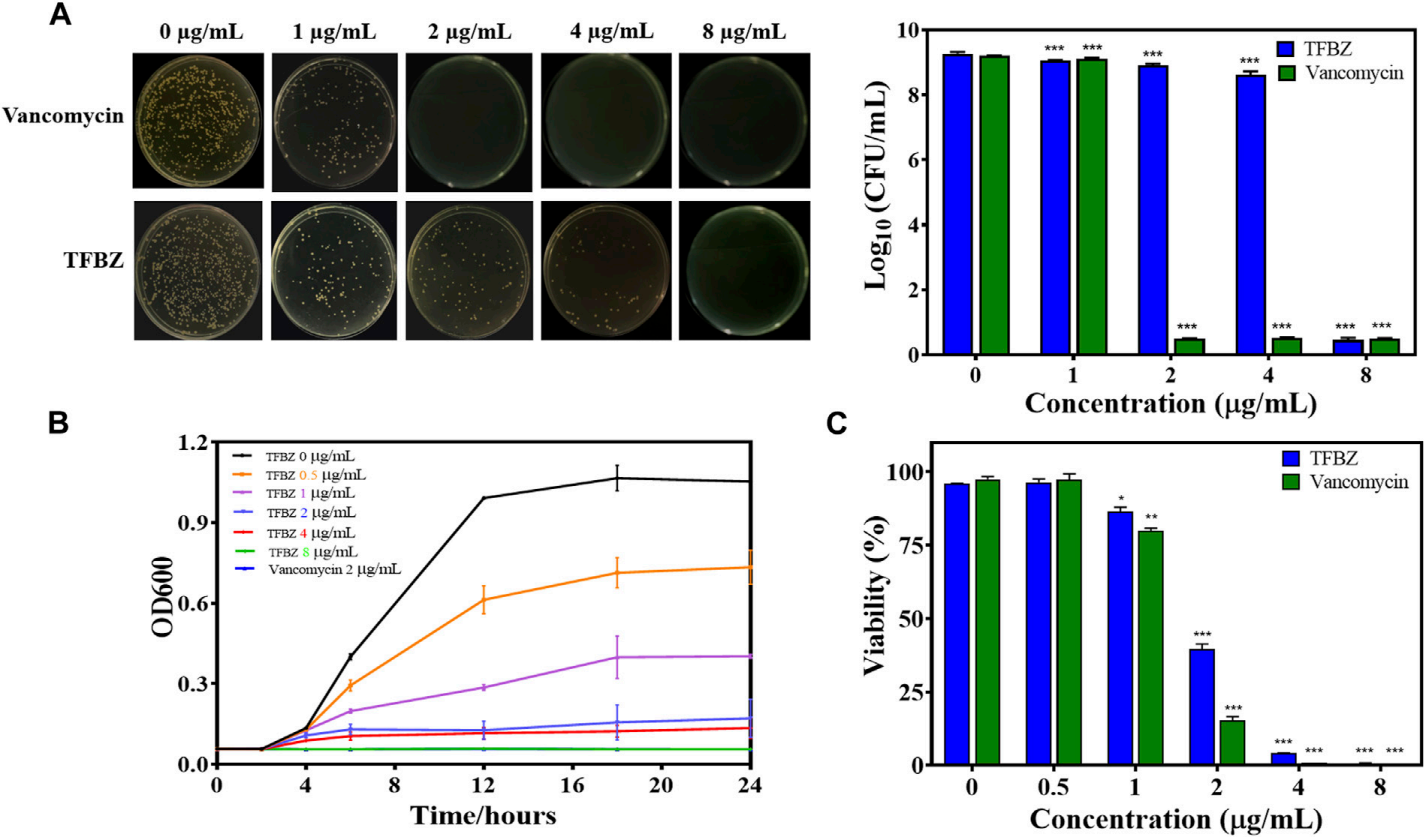
Influence of TFBZ on planktonic MRSA activity. **(A)** Colony photographs and average logarithmic changes of MRSA treated with different concentrations of TFBZ for 24 h. **(B)** Influence of TFBZ on MRSA growth curve dynamics. **(C)** Viability rate of MRSA treated with TFBZ for 24 h. Data are expressed as mean ± SD (*n* = 3). *p < 0.05, **p < 0.01, ***p < 0.001.

To investigate TFBZ antibacterial activity against planktonic MRSA, we performed MIC and MBC assays. As shown in Figure 2A, the colony numbers of MRSA upon exposure to TFBZ were decreased in a dose-dependent manner. According to the visible microorganisms or colony formation, the MIC and MBC value of TFBZ were determined as 4 μg/mL and 8 μg/mL, which was comparable with the positive drug vancomycin (MIC = 2 μg/mL, MBC = 4 μg/mL, Supplementary Table S1). To further explore the bactericidal speed and duration toward MRSA, the time-kill kinetics within 24 h of TFBZ compared to vancomycin were analyzed (Figure 2B). During the first 12 h after administration of 0.5 and 1 μg/mL TFBZ, the growth of MRSA showed an upward trend. Whereas at a concentration of 8 μg/mL, TFBZ exhibited a remarkably fast killing rate and the efficacy were comparable to 2 μg/mL of vancomycin. The turbidity value did not continuously increase with the passage of time, indicating that the sample could not only inhibit the growth rate of bacteria, but also delay the time to enter the logarithmic phase of growth (Du et al., 2020). At the end of 24-h incubation, the anti-MRSA activity of TFBZ was also evaluated by MTT assay (Figure 2C). The inhibitory effect of TFBZ on planktonic MRSA was positively correlated with concentration. TFBZ (4 μg/mL) could decrease the survival rate of MRSA by 95.73%, which was also consistent with MIC value, indicating that TFBZ could significantly affect the metabolic activity of MRSA.

Besides, the antibacterial spectrum of TFBZ against a panel of pathogenic bacteria, including *S. aureus*, MRSA, *P. aeruginosa*, *Salmonella* and *E. coli*, were determined (Supplementary Table S2). Notably, TFBZ could potently inhibit the growth of both susceptible *S. aureus* and drug-resistant MRSA, with MIC_50_ of 0.99 and 1.15 μg/mL, respectively. However, it exhibited minimal antimicrobial activity toward Gram-negative bacteria (*P. aeruginosa*, *Salmonella* and *E. coli)*, with less than 50% reduction even at a high dose of 64 μg/mL.

The antibacterial activity of TFBZ against MRSA was further demonstrated by Live/Dead staining (Supplementary Figure S1A). The green-fluorescent dye (SYTO9) could permeate freely through all the bacterial cell membranes, whereas the red-fluorescent PI penetrates only the dead bacterial membranes (Yu et al., 2022). As expected, almost all the bacteria in the control group exhibited intensive green fluorescence, while a large area of red fluorescence appeared in bacteria upon treated with 4 and 8 μg/mL of TFBZ. Since the cell membrane integrity is damaged, macromolecular substances such as proteins, nucleotides and other intracellular components will reduce, leading to gradual interruption of the cell metabolism (Kang et al., 2019). The protein leakage of MRSA upon exposure to TFBZ was determined by BCA quantifying. As described in Supplementary Figure S1B, the protein in the cell-free supernatant exposed to an increased TFBZ concentration (0–4 μg/mL) exhibited a persistent, significant and dose-dependent leakage within 12 h (Liu et al., 2023) These results indicated that TFBZ could disrupt the integrity of cell membrane, and destruct the growth and metabolic balance of MRSA, which ultimately contributing to bacterial death.

### 3.2 Elimination effect of TFBZ on mature MRSA biofilms

**FIGURE 3:**
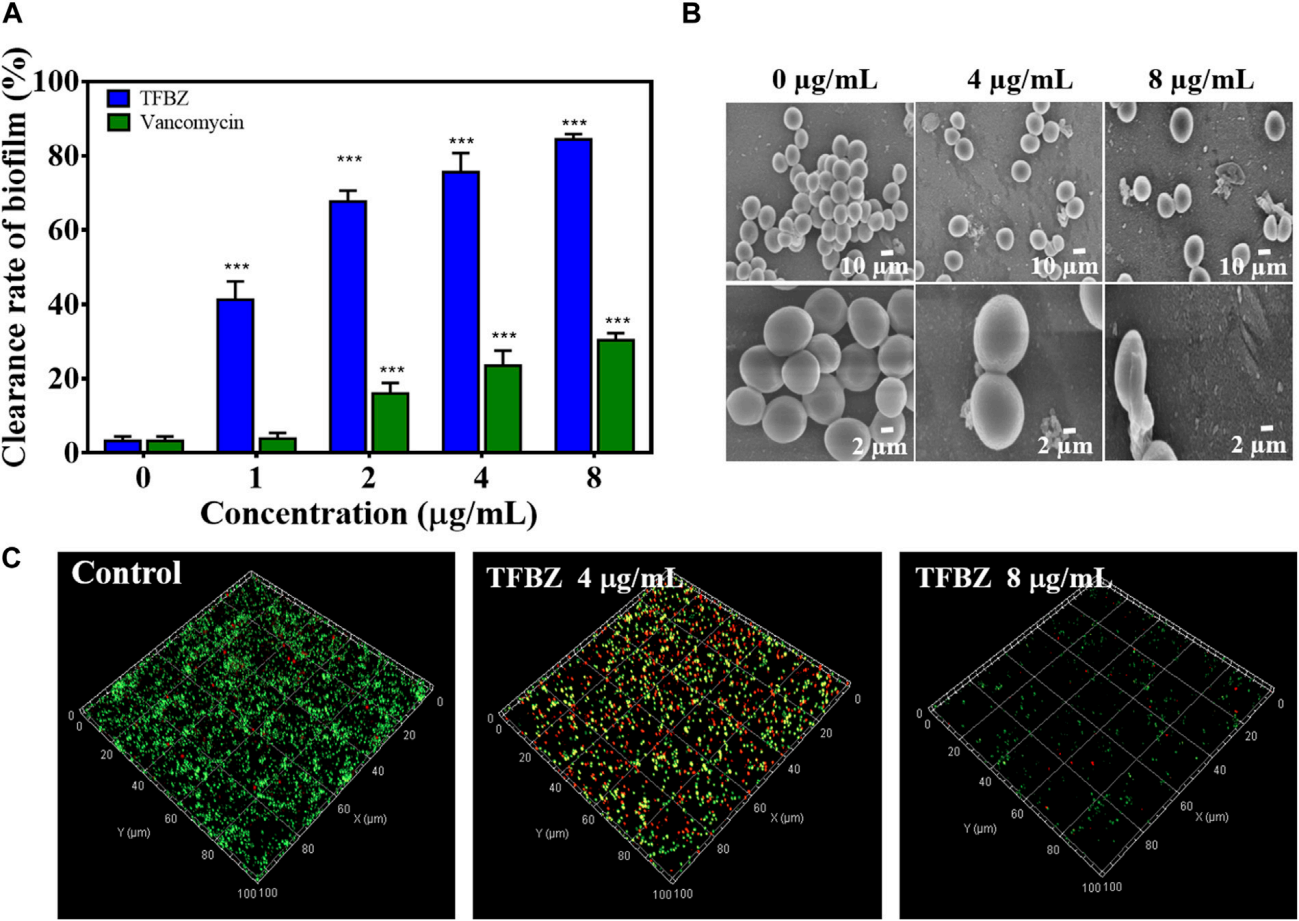
Eradication effect of TFBZ on mature MRSA biofilms. **(A)** Clearance of MRSA biofilms with different concentrations of TFBZ or vancomycin (0, 1, 2, 4, and 8 μg/mL) for 24 h. Data are expressed as mean ± SD (*n* = 3). ***p < 0.001. **(B,C)** SEM **(B)** and CLSM **(C)** images of MRSA biofilms after treatment with TFBZ (0, 4, and 8 μg/mL) for 4 h.

Bacterial biofilms were a three-dimensional community of bacterial cells that adhere to either biotic or abiotic surfaces, and it is aggregates of microorganisms surrounded by extracellular polymeric substances (EPS), contributing to the cumulative tolerance or resistance of bacterial to antibiotics (Tao et al., 2021). Based on these, the ability of TFBZ to disperse preformed biofilm of MRSA was evaluated using the biofilm morphology minimum biofilm eradication concentration (MBEC). Biofilms were established on CBD pegs, and then the pegs were soaked in wells containing TFBZ solution. According to the CBD assay, the relative killing dynamics for TFBZ against both planktonic and biofilm cells can be determined, and the values of MBEC and MBC measured were both 8 μg/mL (Supplementary Table S1), which was consistent with the results from microtiter broth dilution testing. Visualization of biofilms after staining with crystal violet (Figure 3A) indicated that TFBZ produced potent dose-dependent clearance effect on mature biofilms, which was superior to the equivalent concentration of vancomycin (MBEC >32 μg/mL).

Since effective biofilm penetration and retention are the preconditions for TFBZ to kill bacteria deeply, SEM and CLSM were utilized to further observe the effect of TFBZ on the mature biofilms morphology (Antonoplis et al., 2018). As illustrated in Figure 3B, MRSA cells in the control group were uniform with regular sphericity and smooth surface. By contrast, the adhesion and aggregation of biofilms were destructed in TFBZ-treated group, accompanied by surfaces collapse and contents exudation of bacterial cells (Qu et al., 2020). The degree of fluorescence staining on 3D scanning of CLSM reflects the number of bacteria and the thickness of the biofilms (Figure 3C). When MRSA biofilms were exposed to the suspensions of TFBZ (4 μg/mL), the proportion of dead cells (red fluorescence) increased significantly and the biofilms became thinner and looser compared with control, manifesting the accelerated death of MRSA. Both red and green fluorescence were extremely weak in 8 μg/mL TFBZ-treated group, indicating that most of the biofilms had been disrupted. Taken together, these phenomena verified that TFBZ could change permeability and integrity of bacterial cell membrane, leading to efficient eradication of the established MRSA biofilms (Yu et al., 2023).

### 3.3 Effects of TFBZ on bacterial invasiveness and mammalian cell viability

**FIGURE 4:**
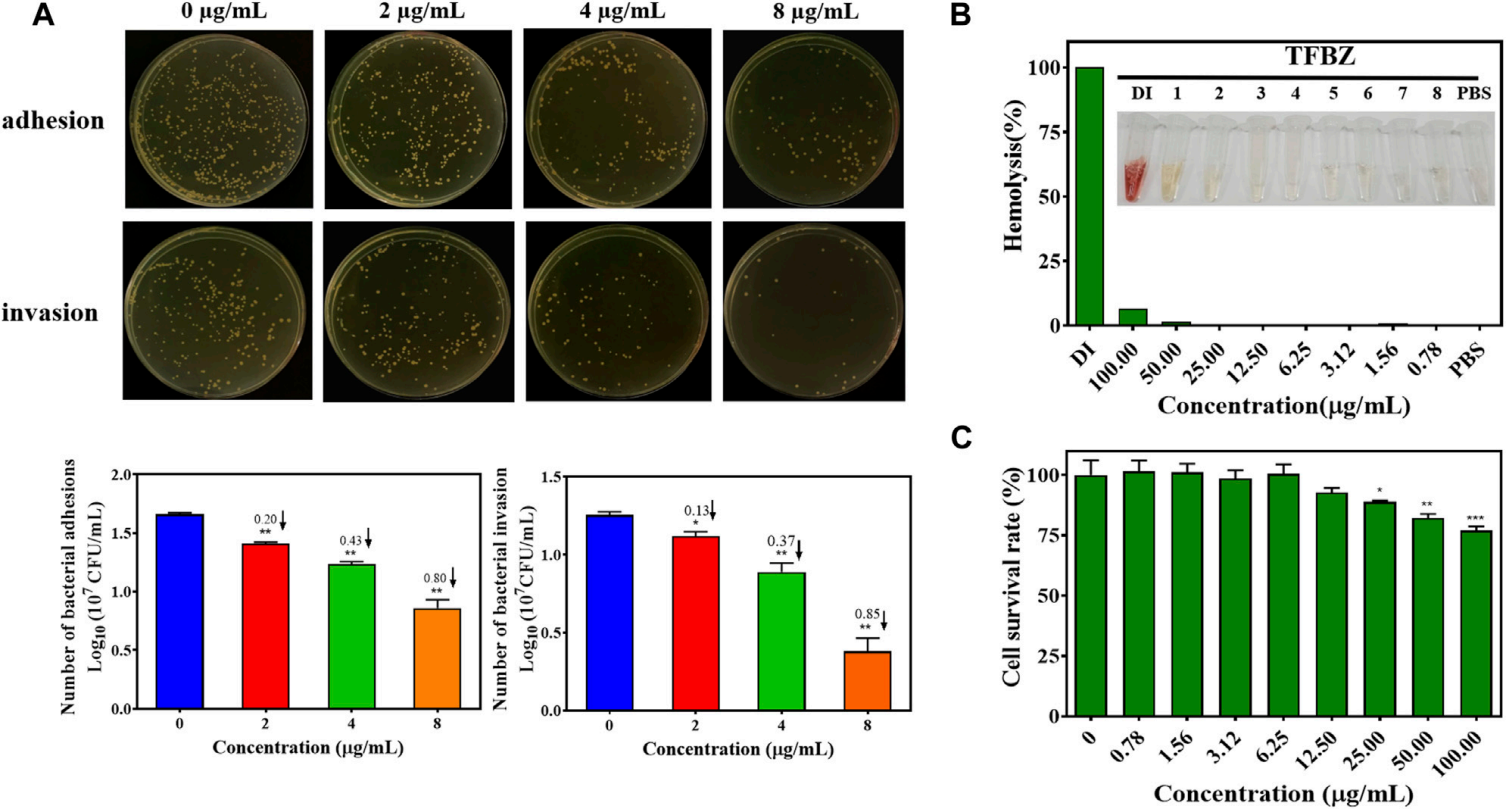
Effects of TFBZ on bacterial invasiveness and mammalian cell viability. **(A)** Colony photographs and average logarithmic changes of MRSA adhering to and invading into MCF-7 cells upon treatment with different concentrations of TFBZ (0, 2, 4, and 8 μg/mL) for 2 h. **(B)** Photographs and hemolysis rate of rabbit whole blood after incubation with different concentrations of TFBZ (0.78–100 μg/mL) for 2 h. **(C)** Cell viability of COS7 cells after incubation with different concentrations of TFBZ (0.78–100 μg/mL) for 24 h. Data are expressed as mean ± SD (*n* = 3). *p < 0.05, **p < 0.01, ***p < 0.001.

Bacterial adhesion and invasion of MCF-7 cells during exposure to TFBZ were quantified by dilution coating plate technique (Figure 4A). Strikingly, upon TFBZ treatment (2, 4, and 8 μg/mL), the initial adhesion of MRSA to MCF-7 cells were significantly suppressed, and the number of adhered MRSA compared with the control group decreased by 0.20, 0.43, and 0.80 Log10 CFU/mL, respectively. Meanwhile, the infection capacity of MRSA also inhibited by TFBZ in a concentration-dependent manner, the bacterial invasion amount decreased by 0.85 Log10 CFU/mL under cultivation with 8 μg/mL TFBZ.

Taking into consideration that biological safety was an important property of antibacterial agents, hemotoxic and cytotoxic experiments were conducted separately to assess the biological safety of TFBZ. As shown in Figure 4B, TFBZ displayed no hemolytic activity toward rabbit red blood cells at the MIC, and still very low level (6.25%) of hemolysis at the concentration as high as 100 μg/mL (25 × MIC). Similarly, Figure 4C shows that TFBZ had no significant cytotoxicity toward COS-7 cells from African green monkey at 12.5 μg/mL (3 × MIC), and also a cell survival rate more than 75% at the highest tested concentration (100 μg/mL). These results elucidated that the benzimidazole derivative TFBZ might not exert bactericidal effect through non-specific membrane-lysing mechanism, moreover, it was biocompatible and propitious to *in vivo* therapeutical application as potential antimicrobial agents (Zhang A. et al., 2023).

### 3.4 Therapeutic effect of TFBZ on MRSA-infectious mice

**FIGURE 5:**
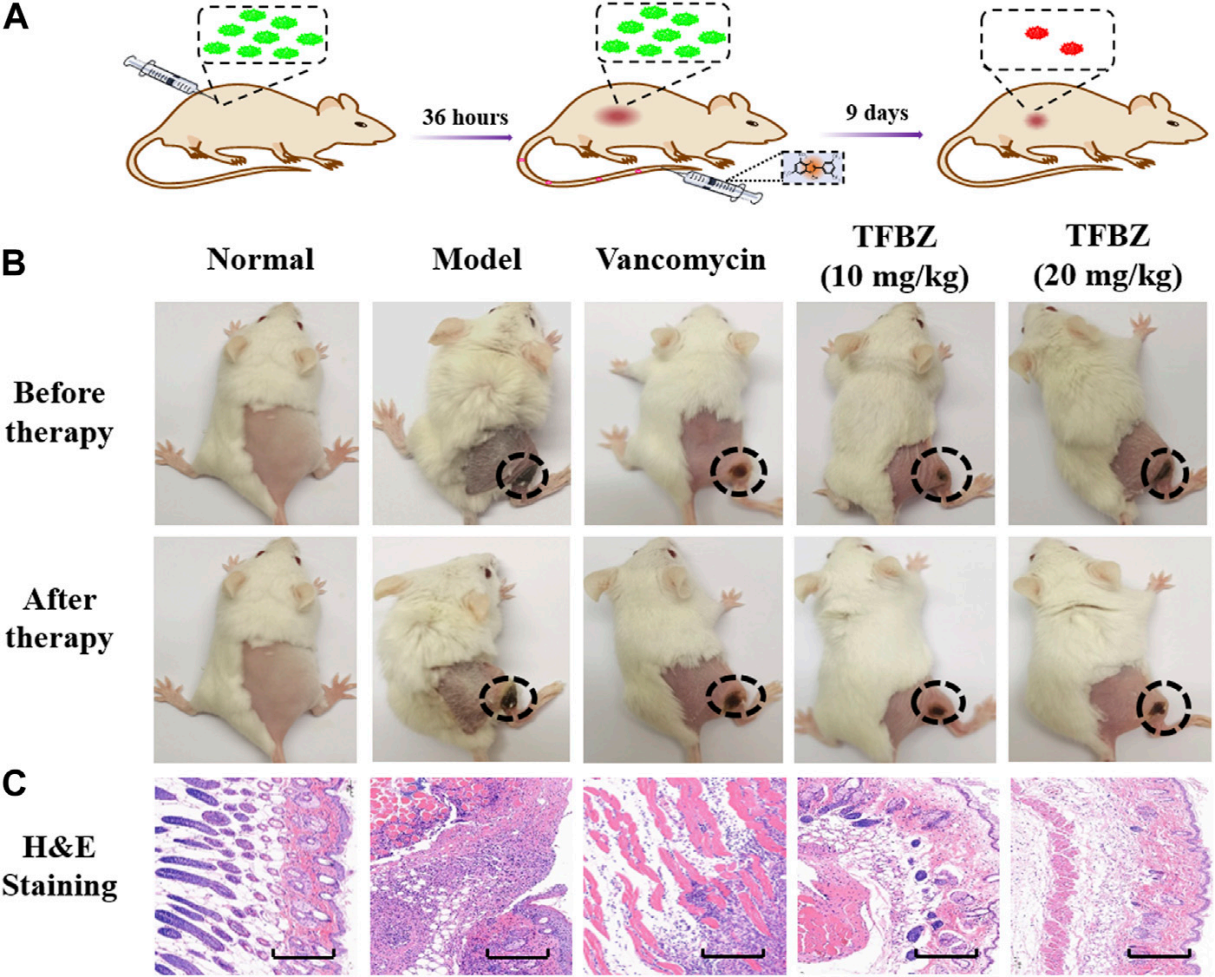
**(A)** Timeline and course of treatment with TFBZ in mice infected with MRSA. **(B)** Typical photographs of infected mice before and after 9 days of treatment by PBS, Vancomycin, TFBZ (10 mg/kg) and TFBZ (20 mg/kg). **(C)** H&E staining images of the infected tissues with different treatments. Scale bar is 10 μm.

**FIGURE 6:**
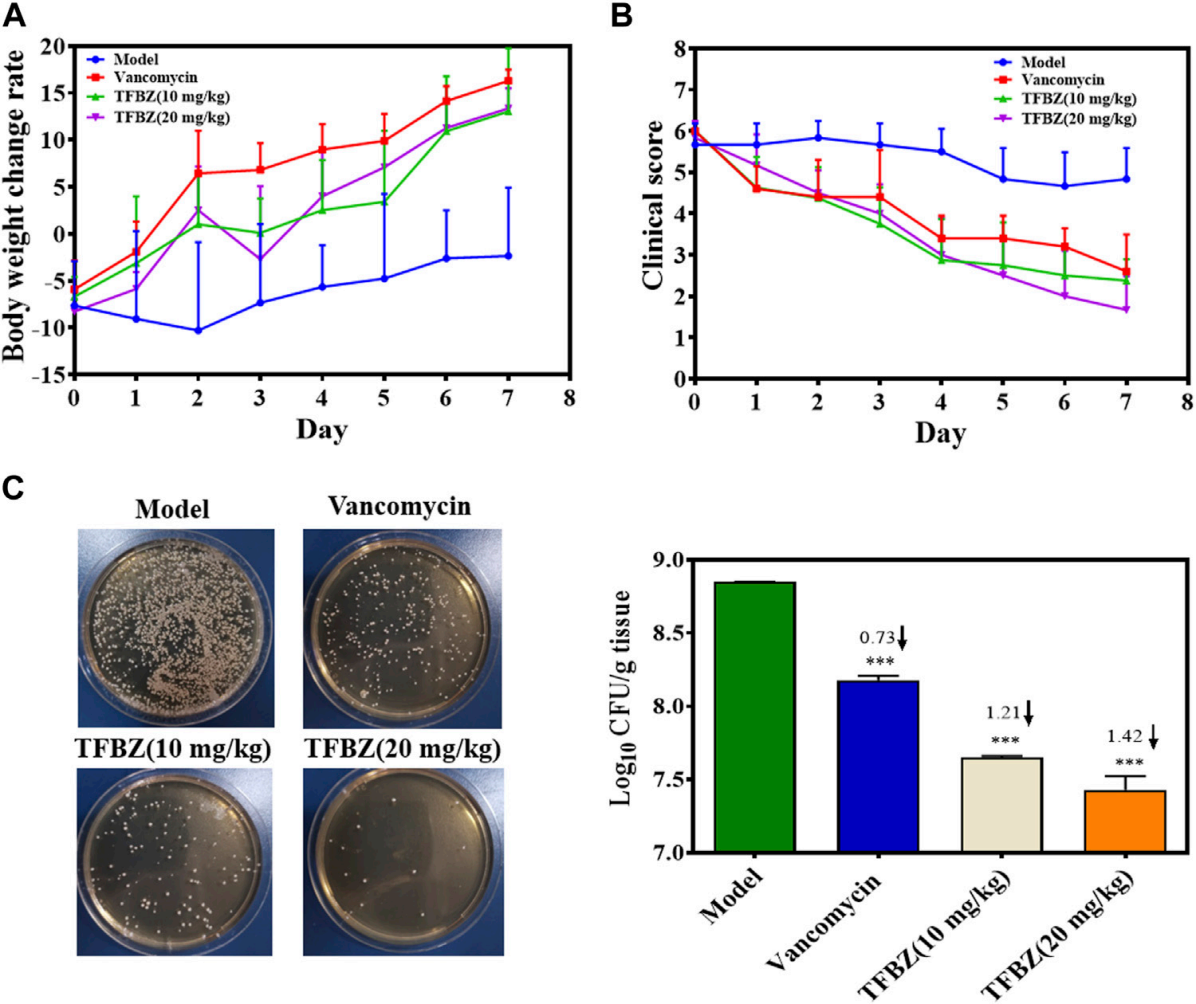
**(A)** Curves of body weight change rate (%) of mice in various group during therapy (*n* = 6). **(B)** Clinical scores of mice after 11 days of treatment (*n* = 6). **(C)** The TSB agar of aliquots from the diluted homogenized infected tissue and average logarithmic changes in bacterial counts after therapy (*n* = 3). Data are expressed as mean ± SD.

Based on the superior antibacterial and biofilm eradication activities of TFBZ *in vitro*, a mouse pyomyositis model was utilized to further explore the therapeutic effectiveness on MRSA biofilm infection. The detailed therapeutic timelines are illustrated in Figure 5A. During the early stages of infection, mice in the model group experienced a significant decrease in body weight. Conversely, mice treated with TFBZ or vancomycin not only rapidly recovered from this weight decline but also steadily gained weight after the infection (Figure 6A). Meanwhile, the clinical scores were also determined daily in Figure 6B. Mice in the model group exhibited a rapid progression of symptoms, characterized by ruffled fur, hind limb degradation and weakness. In contrast, both vancomycin and TFBZ effectively arrested the dissemination of bacteria and recovered hind limb functionality, leading to significant reduction of clinical scores.

Moreover, the representative photographs of the mice pre- and post-treatment are shown in Figure 5B. Obviously before therapy, large dermonecrotic area and abscess volume indicated the extensive tissue infections of MRSA and acute inflammatory response in all the groups. Upon treatment, the infection sites in both the vancomycin and TFBZ groups exhibited predominant scab and healing, distinguishing from the tissue abscesses and wound ulceration in the model group. Furthermore, *in vivo* therapeutic effects were assessed by H&E staining of the infected sites (Figure 5C). In the standard healthy group, the epidermal layer was cohesive, rich in collagen fibers, with clear hair follicles and sebaceous glands (Cao et al., 2020), while the model group exhibited epidermal thickening, dermal hyperplasia, and inflammatory cell infiltration. Nevertheless, the skin tissue structures were more intact in TFBZ-treated group when compared to the vancomycin-treated group, confirming that TFBZ could significantly relieve inflammation and promote wound recovery and skin regeneration. To further verify the efficacy of TFBZ against MRSA biofilm-infection, the bioburden in the infected tissue was quantified using a standard bacterial culture method (Guo et al., 2020) (Figure 6C).In comparison to the model group, both the low dose (10 mg/kg TFBZ) and high-dose (20 mg/kg TFBZ) groups were observed remarkable suppression of MRSA colonies at the infection site, with average log10 reductions of 1.21 and 1.42, respectively. This level of reduction surpassed that achieved by vancomycin treatment, which showed only a log10 reduction of 0.73. Besides, H&E staining was conducted on the major organs, including the heart, lungs, liver, kidneys, and spleen, to exclude obvious histopathological lesions or any potential systemic toxicity (Supplementary Figure S2). Given all above, TFBZ not only greatly improved the dermapostasis caused by MRSA biofilms, but also had desirable biocompatibility.

### 3.5 Expression regulation of TFBZ on genes associated with the survival of MRSA biofilms

**FIGURE 7:**
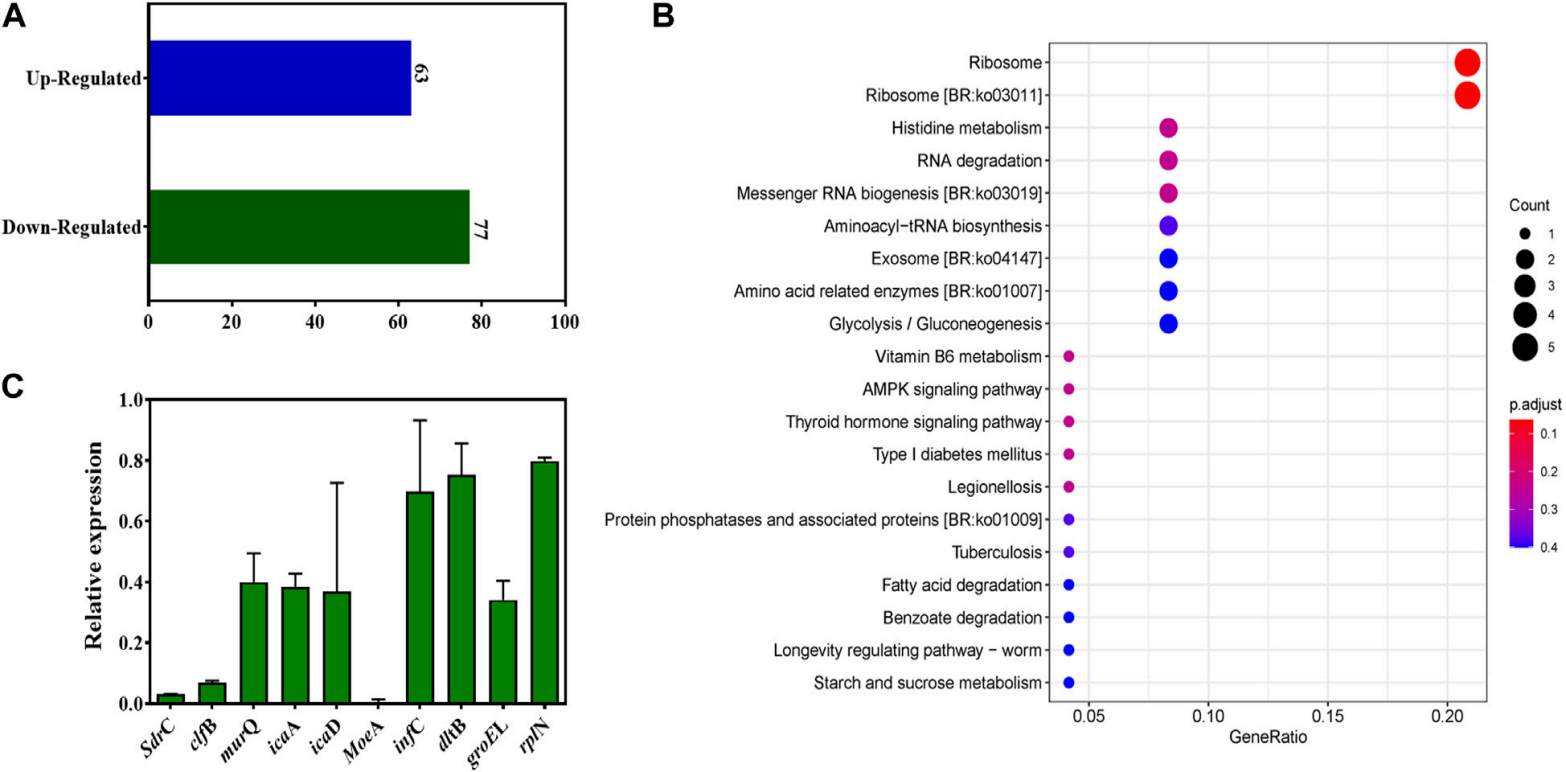
Changes in the transcriptome of MRSA biofilms upon TFBZ treatment and qRT-PCR validation. **(A)** Statistical diagram of differentially expressed genes (DEGs) in the transcriptome of MRSA biofilms between control and TFBZ treatment. **(B)** Top 20 KEGG enrichment pathways for DEGs. **(C)** Relative expression levels of representative genes through real-time quantitative PCR. Data are expressed as mean ± SD (*n* = 3).

To elucidate extensive mechanistic and biological relation regarding TFBZ and biofilms viability, we identified MRSA ATCC 43300 biofilm genes that were differentially expressed upon exposure to TFBZ at 1/4 MIC level through transcript profiling using RNA-seq. By employing a stringent threshold of (*p* < 0.05 and |log2fold change| > 1), a total of 140 differentially expressed genes (DEGs) were screened, among which 63 genes exhibited upregulated expression and 77 genes showed downregulated expression in response to TFBZ treatment (Figure 7A). Furthermore, KEGG enrichment analysis was exploited to reveal the functional information and potential impact on biological pathways of these DEGs. As illustrated in Figure 7B, the ribosome (ko03010) pathway emerged as the most enriched, followed by other significant pathways including histidine metabolism (ko00340), RNA degradation (ko03018), and messenger RNA biogenesis (ko03019).

To validate the observed gene expression regulation of TFBZ in our transcriptome profiling data, quantitative real-time PCR (qRT-PCR) analysis was conducted on nine representative DEGs crucially involved in various biological processes (Figure 7C), including cell adhesion (*sdrC, clfB*), biofilm formation (*icaD, icaA*), coenzyme synthesis (*moeA*), ribosomal protein (*rplN*), protein synthesis (*infC*), and cell wall biosynthesis (*murQ, dltB*). As expected, the results consistently showed pattern-like downregulation of these selected DEGs, suggesting that TFBZ exerted multi-targeted inhibitory effect on MRSA survival and biofilm formation. In this study, TFBZ decreased the expression of *sdrC* and *clfB*, interfering with the adhesion and aggregation of MRSA on host cells or solid surface, thus preventing the initial formation of the biofilms (Foster et al., 2014; Kang et al., 2022). Meanwhile, downregulation of *icaA* and *icaD* genes derived from TFBZ treatment reduced the production of polysaccharide intercellular adhesin, thereby interfering the stability and motility of the bacterial biofilms (Hernández-Cuellar et al., 2023). Furthermore, TFBZ interfered with the expression of translation initiation factor-*infC* and ribosomal protein*-rplN*, attributing to impediment of key proteins synthesis required for microbial growth and biofilm formation (Korostelev et al., 2010). Additionally, downregulation of *murQ* and *dltB* genes hindered the biosynthesis of peptidoglycan precursors and affected the remodeling of the cell wall, reducing the resistance of MRSA biofilms toward the host immune system and antibiotics (Wood et al., 2018; Ma et al., 2022). Since *MoeA* protein is essential for production of active molybdoenzymes, TFBZ might perturb the assemble of molybdenum cofactor into the molybdoenzymes, leading to the metabolism disorder of nitrate reduction and sulfur compound conversion in MRSA (Hasona et al., 1998). Collectively, these findings imply that the TFBZ compound may eradicate MRSA biofilms through affecting various molecular pathways, which potentially reducing the risk of bacterial resistance to drugs.

## 4 Conclusion

In summary, we have illustrated that this fluorinated benzimidazole derivative TFBZ displayed potent antibacterial activity against both planktonic and persistent MRSA. Moreover, it exhibited robust ability to eliminate bacterial burden and reduce abscess lesions in murine subcutaneous abscess infection model. Subinhibitory concentration of TFBZ could effectively disrupt MRSA biofilms through transcriptionally regulating the gene expression involving cell adhesion, biofilm formation, translation process, cell wall biosynthesis. These rapid killing kinetics, robust anti-biofilms characteristics, gentle biocompatibility and low tendency to develop drug resistance highlight that the compound TFBZ merits further development as a novel antimicrobial.

## Data Availability

The datasets presented in this study can be found in online repositories. The names of the repository/repositories and accession number(s) can be found below: https://www.ncbi.nlm.nih.gov/sra/PRJNA1043129, PRJNA1043129.
