# Supplementary material for: Bactericidal and biofilm eradication efficacy of a fluorinated benzimidazole derivative, TFBZ, against methicillin-resistant Staphylococcus aureus
Source: Front Pharmacol. 2024 Apr 10;15:1342821. doi: 10.3389/fphar.2024.1342821 (PMC11039886; doi:10.3389/fphar.2024.1342821)
Supplement: Supplementary file 1 [file DataSheet1.docx]

Supplementary Material

Bactericidal and biofilm eradication efficacy of a fluorinated benzimidazole derivative, TFBZ, against methicillin-resistant *Staphylococcus aureus*

**Qian Chen^1†^，** **Zhihui Dong^1†^，** **Xuedi Yao^1†^， Huan Sun^1^， Xin Pan^2^*， Jikai Liu^1^* and Rong Huang^1^*******

*** Correspondence:** Xin Pan, [px520520@126.com](mailto:px520520@126.com); Jikai Liu, [liujikai@mail.scuec.edu.cn](mailto:liujikai@mail.scuec.edu.cn); Rong Huang, [ronghuang@mail.scuec.edu.cn](mailto:ronghuang@mail.scuec.edu.cn).

**Table of Contents**

[1 ^1^H NMR, ^13^C NMR and ^19^F NMR spectra of TFBZ 2](#_Toc162362453)

[2 High-resolution mass spectra of TFBZ 4](#_Toc162362454)

[3 Supplementary Figures and Tables 5](#_Toc162362455)

[3.1 Supplementary Tables 5](#_Toc162362456)

[3.2 Supplementary Figures 6](#_Toc162362457)

# ^1^H NMR, ^13^C NMR and ^19^F NMR spectra of TFBZ

^1^H NMR (500 MHz, DMSO-*d*_6_) of **TFBZ**

^13^C NMR (125 MHz, DMSO-*d*_6_) of **TFBZ**

^19^F NMR (471 MHz, DMSO-*d*_6_) of **TFBZ**

# High-resolution mass spectra of TFBZ

High-resolution mass spectrum of **TFBZ**


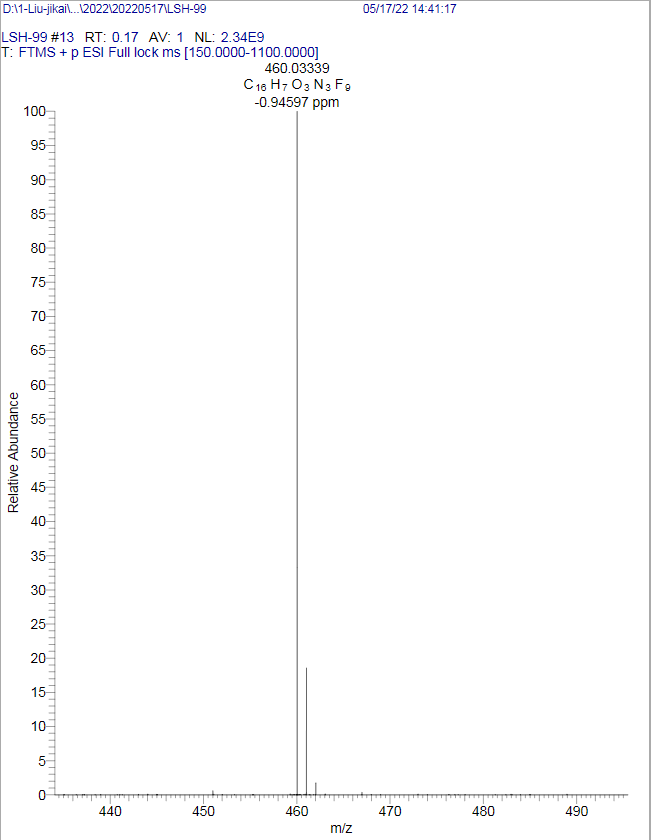


# Supplementary Figures and Tables

## Supplementary Tables

**Supplementary Table 1.** Anti-MRSA activity of TFBZ and vancomycin

| Compound | MIC(µg/mL) | MBC(µg/mL) | MBEC(µg/mL) |
| --- | --- | --- | --- |
| TFBZ | 4 | 8 | 8 |
| Vancomycin | 2 | 4 | - |

^a^ MIC represents minimum inhibitory concentration.

^b^ MBC represents minimum bactericidal concentration.

^C^ MBEC represents minimum biofilm eradication concentration.

**Supplementary Table 2.** Antibacterial effect of TFBZ against a series of strain

| Bacterial strains | MIC_50_ (μg/mL) |
| --- | --- |
| MRSA | 1.15 |
| *S. aureus* | 0.99 |
| *E. coli* | 42.95 |
| *P. aeruginosa* | >64 |
| *Salmonella* | >64 |

^a^ MIC_50_ represents the minimum inhibitory concentration required to inhibit the growth of 50% of a specific microbial population.

**Supplementary Table 3.** RT-qPCR Primer sequences

| Gene | Primer sequences (5´to 3´) |
| --- | --- |
| *SdrC* | Forward: CCTGCTGGCTTAACACAA  Reverse: AGTCGCTATCTGAGTCTGA |
| *ClfB* | Forward: GGAAGTGCTGATGGTGATT  Reverse: TCTGAGTCTGAGTCGCTAT |
| *infC* | Forward: CGGTCAAGATGGTGAACAA  Reverse: ACTGGTGGTTTCGCATTC |
| *dltB* | Forward: GGTTAGGTGGACATCAGATT  Reverse: ACGATATTCATTGCCTGTTG |
| *MurQ* | Forward: GTACTTCTGGTGCTAATGATAC  Reverse: ACGCCATTGCTTCATCAT |
| *MoeA* | Forward: AATCAGGTGCGGTTGTAG  Reverse: CAGCGTCAGATTCAGTCA |
| *rplN* | Forward: CAGGTGTTCGTCGTAATGA  Reverse: GTACTTCTGGTGCTAATGATAC |
| *icaD* | Forward: ATGGTCAAGCCCAGACAGAG  Reverse: AGTATTTTCAATGTTTAAAGCAA |
| *icaA* | Forward: ACACTTGCTGGCGCAGTCAA  Reverse: TCTGGAACCAACATCCAACA |
| *16S rDNA* | Forward: CGCAATGGGCGAAAGC  Reverse: TACGATCCGAAGACCTTCATCA |

## Supplementary Figures


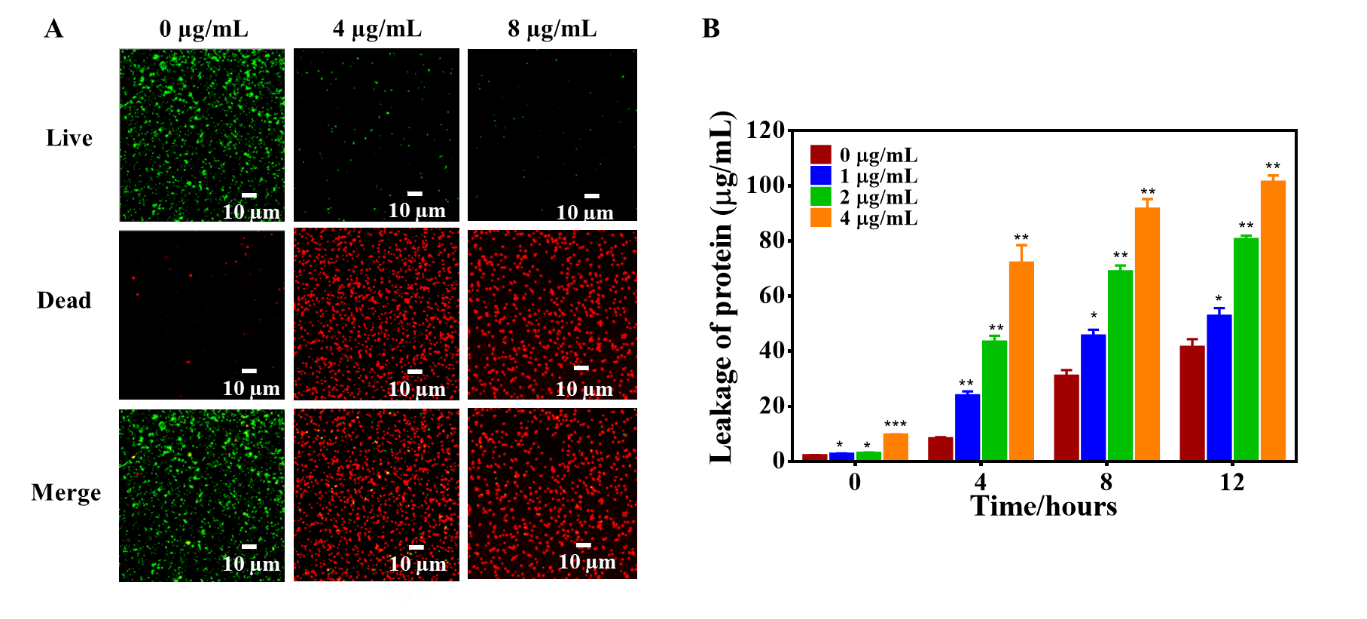
**Supplementary Figure 1.** Effect of TFBZ on planktonic MRSA. **(A**) Confocal images of MRSA stained with SYTO9 (green) and PI (red) after incubation with TFBZ (0, 4, 8 μg/mL) for 2 h**. (B)** Protein leakage of MRSA upon exposure to TFBZ (0, 1, 2, 4 μg/mL) on at different time intervals (0, 4, 8 and 12 h).


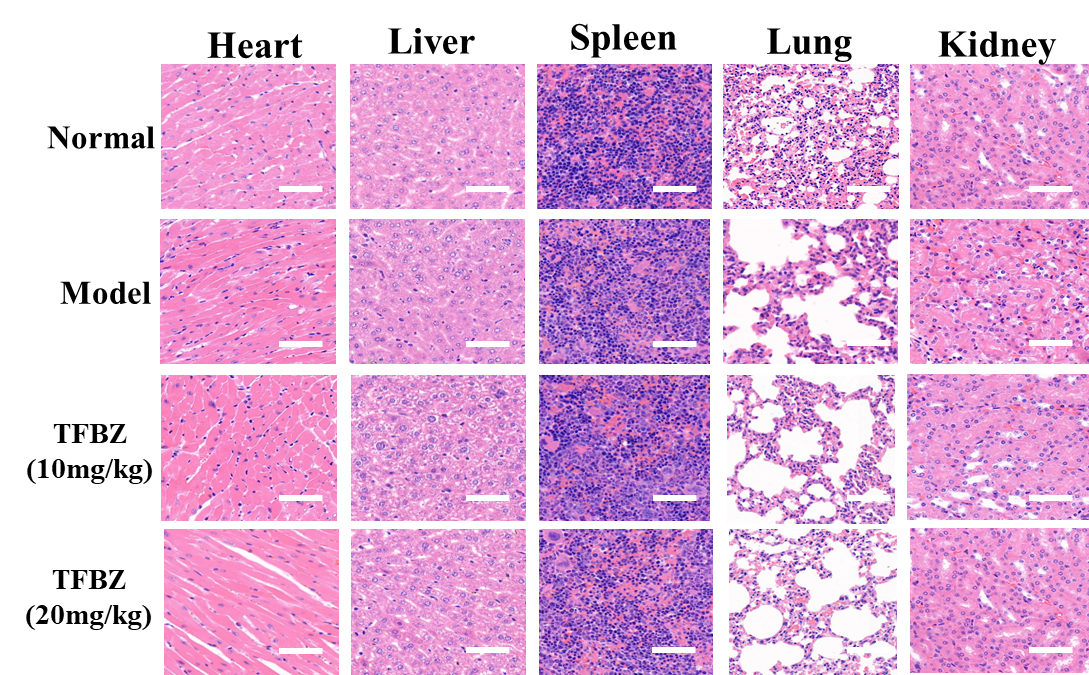


**Supplementary Figure 2.** Representative H&E staining images of the main organs (heart, liver, spleen, lung, and kidney) collected from mice sacrificed after therapy.
